# Toll-Like Receptors Signaling Pathway of Quercetin Regulating Avian Beta-Defensin in the Ileum of Broilers

**DOI:** 10.3389/fcell.2022.816771

**Published:** 2022-02-23

**Authors:** Linlin Ying, Hao Wu, Shuaishuai Zhou, Han Lu, Manyi Ding, Bo Wang, Shanshan Wang, Yanjun Mao, Fenglin Xiao, Yao Li

**Affiliations:** Institute of Animal Nutrition, Northeast Agricultural University, Harbin, China

**Keywords:** quercetin, avian beta-defensin, Toll-like receptor, ileum, broilers

## Abstract

The purpose of the experiment was to investigate the Toll-like receptor signaling pathway of quercetin regulating avian beta-defensin (AvBD) in the ileum of Arbor Acre (AA) broilers. Four hundred and eighty one-day-old Arbor Acre broilers with similar body weight, half male and female, were randomly allotted to four treatments; the control treatment and three dietary treatments were fed with the basal diets supplemented with 0, 0.02%, 0.04, and 0.06% quercetin, respectively. The results showed that dietary quercetin supplementation did not significantly influence growth performance (*p* > 0.05), but significantly decreased the mortality rate of broilers by 85.74%, 85.74, and 71.28%, respectively (*p* < 0.05, F = 9.06). Compared with control, dietary supplementation with 0.04 and 0.06% quercetin significantly upregulated mRNA expression of total AvBD (*p* < 0.05), and there were no significant differences in the mRNA expression of AvBD1, AvBD2, and AvBD14 in three quercetin supplementation groups in the ileum of AA broilers (*p* > 0.05). Dietary supplementation with 0.02 and 0.06% quercetin significantly downregulated the mRNA expression of total Toll-like receptors (*p* < 0.05). Dietary quercetin supplementation significantly downregulated the mRNA expression of TLR1A, TLR1B, and TLR2A (*p* < 0.05); however, there were no significant differences in the mRNA expression of TLR2B, TLR5, and TLR15 (*p* > 0.05). Dietary quercetin supplementation significantly downregulated the mRNA expression of myeloid differentiation primary response protein 88 (MyD88) and TIR domain-containing adaptor protein/MyD88-adaptor-like (TIRAP/MAL) (*p* < 0.05), 0.02% quercetin significantly downregulated the mRNA expression of tank-binding kinase1 (TBK1), IκB kinase complex-α (IKKα), IKKβ, IKKε, nuclear factor-kappa B (NF-κB), NF-κB inhibitor-alpha (IκBα), IκBα, IκBβ, TNF-receptor-associated factor 3 (TRAF3), and interferons regulatory factor 7 (IRF7) (*p* < 0.05), 0.04% quercetin significantly downregulated the mRNA expression of IKKβ, IKKε, NF-κB, IκBα, IκBβ, TRAF3, and TRAF6 (*p* < 0.05), and 0.06% quercetin significantly downregulated the mRNA expression of TBK1 and IKKα (*p* < 0.05). 0.02% quercetin significantly decreased the relative abundance of *Escherichia*, *Staphylococcus* (*p* < 0.05), and *Salmonella* (*p* < 0.01), 0.04% quercetin significantly decreased the relative abundance of *Staphylococcus* (*p* < 0.05), *Escherichia*, and *Salmonella* (*p* < 0.01), and 0.06% quercetin significantly decreased the relative abundance of *Salmonella* (*p* < 0.05) and *Staphylococcus* (*p* < 0.01) in the ileum of AA broilers. These findings suggested that dietary quercetin supplementation regulated the mRNA expression of AvBD, TLR, and the TLR signaling pathways and NF-κB signalling pathways, thereby maintaining the microecological balance of the intestinal tract and decreasing the mortality of broilers, and the optimum addition amount of quercetin is 0.04% under the test conditions.

## Introduction

The intestine plays a crucial role in maintaining body immunity that is the first line of defense from exogenous pathogens infecting host cells and tissues of chicken, which is also the largest organ with immune properties (Fisinin and Surai, 2013). Toll-like receptors (TLRs) are evolutionarily conserved pattern recognition receptors that have been identified in chickens, which initiate innate immune responses and regulate the expression of proinflammatory and anti-inflammatory cytokines and avian beta-defensin (AvBD) (St Paul et al., 2013; Yoshimura, 2015; Kang, et al., 2019; Shimizu et al., 2020; Terada et al., 2020). TLR-mediated signaling pathways are involved in regulating intestinal epithelial barrier integrity and promoting the health of chickens (Nighot et al., 2017), which leads to a well-studied signaling cascade initiated by TIR domain-containing signaling adapters, including myeloid differentiation primary response protein 88 (MyD88), TIR domain-containing adaptor inducing IFN-β (TRIF), TIR domain-containing adaptor protein/MyD88-adaptor-like (TIRAP/MAL), and TRIF-related adaptor molecule (TRAM), and these adapters interact with the TIR domain of the TLR (Hamerman et al., 2015). Administration of TLR-Ls alone also may prevent bacterial infections in chickens (Gomis et al., 2003; Taghavi et al., 2008). β-defensin found in birds is an important and versatile component of the innate immune system in all species and belongs to a kind of the antimicrobial peptides (Lynn et al., 2007), which are produced by phagocytic cells, leukocytes, and epithelial cells, has bactericidal activity against a variety of Gram-negative and Gram-positive bacteria, may directly act on immune cells or form a complex with other molecules to regulate immune responses, and controls the interaction between the host and microorganism, thus maintaining host health and reducing the burden of infectious diseases (Mahlapuu et al., 2016; Hall et al., 2017; Meade and O'Farrelly, 2019). Daneshmand et al. (2020) reported that antimicrobial peptides improve the intestinal damage associated with necrotic enteritis, reduce mortality, and restore the microflora balance in the ileum of broilers. The cecum and ileum of broilers are widely concerned, cecum is the most abundant intestinal microflora and the main place for pathogen colonization (Lan et al., 2002), and ileum is the key part of nutrient absorption, with high microbial diversity (Tang et al., 2000). There are few studies related to quercetin, AvBD, and TLRs in the ileum of broilers. It is shown that flavonoids can modify the composition of the microbiota, modulate TLR gene expression, and regulate the TLR-mediated signaling pathways (Pérez-Cano et al., 2014). Quercetin, a major flavonoid, has been indorsed for its antioxidant and anti-inflammatory properties (Comalada et al., 2006; Saeed et al., 2017). The present study was undertaken to confirm the relationship between mortality and AvBD in the ileum of Arbor Acre (**AA**) broilers. In addition, the effects of quercetin on TLR and signal molecules related to the TLR signaling pathway were explored to elucidate the mechanism by which dietary quercetin supplementation in AA broilers improves health.

## Materials and Methods

All procedures used in this study were approved by the Animal Welfare Care and Use Committee of the University. Housing, management, and care of the birds conformed to the guidelines of Agricultural Animal in Agricultural Research and Teaching of Heilongjiang Province (HEI Animal Management Certificate No. 11928).

### Materials

Main reagents: quercetin (purity ≥97%) was purchased commercially from Sigma-Aldrich chemical company, United States.

Animals: 1-day-old AA broilers were purchased from Harbin Yinong Poultry Industry, China.

### Experimental Design

All the broilers were individually weighed; 480 broilers (1 day old) with similar body weight were randomly divided into four groups with six replicates, with 20 broilers in each replicate (120 broilers per treatment). The assigned groups were as follows: A, normal control broilers fed with basal diet (NC); B, the treatment broilers fed with basal diet of 0.02% quercetin (quercetin 0.02%); C, the treatment broilers fed with basal diet of 0.04% quercetin (quercetin 0.04%); and D, the treatment broilers fed with basal diet of 0.06% quercetin (quercetin 0.06%), and the trial lasted 42 d. Quercetin dehydrate powder was mixed with basal diet and offered in mash form (5mm) after grinding (Table 1).

**TABLE 1:** The experimental design.

Group	Treatment
A (control)	Basal diet
B (quercetin 0.02%)	Basal diet+ 0.02% quercetin
C (quercetin 0.04%)	Basal diet+ 0.04% quercetin
D (quercetin 0.06%)	Basal diet+ 0.04% quercetin

All the broilers were raised under the same management and environment. Continuous fluorescent light was provided during the experiment. Water and experimental diets were available *ad libitum*, followed by routine immunization. At the end of the experiment, two chickens per replicate were randomly selected and slaughtered, and the intestinal tract was excised out, washed in ice-cold normal saline, patted dry and frozen in cryopreserved tubes in liquid nitrogen, and stored at −80°C.

### Experimental Diets

Corn-soybean meal basal diets were formulated according to Chinese Broiler Feeding Standards (GB/T5916-2020) to meet the nutrient requirements of AA broilers (Table 2).

**TABLE 2:** Composition and nutrient level of basal diets.

Composition	% (air-dry basis)	
	1–3 weeks	4–6 weeks
Ingredients		
Corn	60.00	65.80
Soybean meal	28.80	21.40
Soybean oil	2.37	2.73
Fish meal	5.89	8.00
Methionine	0.20	0.07
Dicalcium phosphate	0.94	0.47
Limestone	1.05	0.98
Sodium chloride	0.32	0.15
Premix	0.30	0.30
Choline	0.13	0.10
Total	100.00	100.00
Nutrient level		
ME (MJ/kg)	12.54	12.96
CP (%)	21.50	20.00
Total lysine (%)	1.19	1.11
Met + Cys (%)	0.91	0.76
Ca (%)	1.00	0.90
Total P (%)	0.73	0.65
Available P (%)	0.45	0.40

Provided per kilogram of diet: vitamin A, 10,000 IU; vitamin D_3_, 1250 IU; vitamin E, 25 IU; vitamin K, 1.0 mg; vitamin B_1_, 2.5 mg; vitamin B_2_, 8.0mg; vitamin B_6_, 4.0 mg; vitamin B_12_, 0.015 mg; biotin, 0.20 mg; folic acid, 0.8 mg; pantothenic acid, 12 mg; nicotinic acid, 35 mg; Cu (CuSO_4_·5H_2_O), 10 mg; I (KI), 0.7 mg; Fe (FeSO_4_·7H_2_O), 100 mg; Mn (MnSO_4_·H_2_O), 120 mg; Se (NaSeO_3_), 0.3mg; Zn (ZnO), 100 mg.

### Determination of Indexes

#### Growth Performance

At 0, 21, and 42 days of age, one broiler per replicate was randomly selected from each treatment group and weighed after feed deprivation for 12 h, and growth performance in terms of average daily gain (ADG), average daily feed intake (ADFI), and feed conversion ratio (FCR) per replicate (cage) was recorded and calculated.

#### Mortality Rate

From day 8 of the trail, the mortality rate of AA broilers in each group was recorded and calculated.

#### mRNA Expression of Genes

At the end of the experiment, one broiler per replicate from each treatment group was randomly selected and slaughtered, ileum content was individually homogenized, and total RNA was extracted using TRIzol reagent (Invitrogen Corp., Carlsbad, CA, United States), and cDNA was synthesized using a PrimeScript 1st strand cDNA synthesis kit (Takara Bio Inc., Kyoto, Japan). RT-qPCR was performed using the SYBR Premix Ex Taq II, ROX plus (Takara Bio Inc.) and the Prism 7900HT sequence detection system (Applied Biosystems, Foster City, CA, United States). PCR was carried out for 40 cycles (2 min at 50°C, 10 min at 95°C, and 40 cycles of 10 s at 95°C and 1 min at 60°C) (Seo et al., 2016). β-Actin was used as the internal control in this study. According to the gene bank, the primers were designed by Oligo 6.0 software and Primer premier 5.0 software (Tables 3–5).

**TABLE 3:** Primers of genes related to avian beta-defensins used for mRNA expression.

Target gene	Primer sequences	GenBank accession
β-Actin	F:5′-GCACCACACTTTCTACAATGAG-3′	NM_205518.1
	R:5′-ACGACCAGAGGCATACAGG-3′	
AvBD1	F:5′-CCCTTCATCCTCCTCCTGGC-3′	NM_204993.1
	R:5′-TGATGAGAGTGAGGGAAGGGC-3′	
AvBD2	F:5′-TCCAGGCTTCTCCAGGGTTG-3′	NM_001201399.1
	R:5′-GACACCCTCCAAAGTGGCAG-3′	
AvBD3	F:5′-TGCCACTCAGTGCAGAATAAGAGG-3′	NM_204650.2
	R:5′-CGAGAAGCCACGGCGATGTC-3′	
AvBD4	F:5′-GGCAGTTCATGGAGCTGTGG-3′	NM_001001610.2
	R:5′-AGAAGGTCCCGCGATATCCA-3′	
AvBD5	F:5′-TGCTGTCCTCCTCCTGATGCTC-3′	NM_001001608.2
	R:5′-CGGCAGCAGAAGTCTTCCTTGG-3′	
AvBD6	F:5′-TGTTGCAGGTCAGCCCTACT-3′	NM_001001193.1
	R:5′-CCACCTGTTCCTCACACAGC-3′	
AvBD7	F:5′-CTTTGTGGTGCTCCAGGGTG-3′	NM_001001194.1
	R:5′-ATGGCCTTCGACAGATCCCT-3′	
AvBD8	F:5′-GCGTACCTAACAACGAGGCACAG-3′	NM_001001781.1
	R:5′-AAGGCTCTGGTATGGAGGTGGAAG-3′	
AvBD9	F:5′-GCCAAGAAGACGCTGACACCTTAG-3′	NM_001001611.2
	R:5′-TCAACTGAAGGAGCACGGCATG-3′	
AvBD10	F:5′-TCGCTGTTCTCCTCTTCCTCTTCC-3′	NM_001001609.2
	R:5′-GCGCCGGAATCTTGGCACAG-3′	
AvBD11	F:5′-GGCTCTGCTCCTCTTCCTCCTC-3′	NM_001001779.1
	R:5′-TTCTGACGCCAAGAGCATGTTCC-3′	
AvBD12	F:5′-TTCATCTCCCTGCTCGCTCA-3′	NM_001001607.2
	R:5′-AGGTCTTGGTGGGAGTTGGT-3′	
AvBD13	F:5′-GCCATCGTTGTCATTCTCCTCCTC-3′	NM_001001780.1
	R:5′-AAGCAGAGCCTCCGGCAGTG-3′	
AvBD14	F:5′-CCAGAGTCGGACACTGTCACATG-3′	NM_001348511.1
	R:5′-TTGCCAGTCCATTGTAGCAGGTAC-3′	

**TABLE 4:** Primers of genes related to Toll-like receptors used for mRNA expression.

Target gene	Primer sequences	GenBank accession
β-Actin	F:5′-GCACCACACTTTCTACAATGAG-3′	NM_205518.1
	R:5′-ACGACCAGAGGCATACAGG-3′	
TLR1LA	F:5′-TTACTGCCAATTGCTTGCAC-3′	NM_001007488.4
	R:5′-GGTTAGGAAGACCGTGTCCA-3′	
TLR1LB	F:5′-CCCGTTCAAGTGTTCATGTG-3′	NM_001081709.3
	R:5′-GTTCCGCTCAAGTCTTCTGG-3′	
TLR2A	F:5′-ACATGTGTGAATGGCCTGAA-3′	NM_204278.1
	R:5′-TTGAGAAATGGCAGTTGCAG-3′	
TLR2B	F:5′-TTCGCTCCAACACCTTCG-3′	NM_001161650.1
	R:5′-CTGATGACTGCTGAAATACG-3′	
TLR3	F:5′-AGACACAGCAATTCAGAAC-3′	NM_001011691.3
	R:5′-TTAATGATGTTATTATCCTCCAAG-3′	
TLR4	F:5′-CCATCCACTCAGACAACCTTTCCA-3′	NM_001030693.1
	R:5′-AGTAAACGCAGCAGACGCG-3′	
TLR5	F:5′-CACTGCTGGAGGATTTGTTCTTG-3′	NM_001024586.1
	R:5′-ACAGACGGAGTATGGTCAAACG-3′	
TLR7	F:5′-GATGCAGTGTGGTTTGTTGG-3′	NM_001011688.2
	R:5′-AACCAAGCTCCTCCCTTTGT-3′	
TLR15	F:5′-TCTTCTGGTATCTGGTCTTGC-3′	NM_001037835.1
	R:5′-CCTGGATTGGGTGGATCTTC-3′	
TLR21	F:5′-AGCTGGAGCTGTTGGACCTA-3′	NM_001030558.1
	R:5′-TTCACGTGCCATAGCATCTC-3′	

**TABLE 5:** Primers of genes related to the Toll-like receptors signaling pathway used for mRNA expression.

Target gene^a^	Primer sequences	GenBank accession
MyD88	F:5′-AGAAGGTGTCGGAGGATGGTG-3′	NM_001030962.4
	R:5′-GGGCTCCAAATGCTGACTGC-3′	
TIRAP/MAL	F:5′-CTCATAGCACCACCAGCCACTC-3′	NM_001024829.1
	R:5′-GGGTAATCCTTCCTGTCAATGTCC-3′	
NF-κB	F:5′-TTGCTGCTGGAGTTGATGTC-3′	NM_001001472.2
	R:5′-TGCTATGTGAAGAGGCGTTG-3′	
IκBα	F:5′-CTTCCAGAACAACCTCAGCCAGAC-3′	NM_001001472.2
	R:5′-CGCAGCCAGCCTTCAGCAG-3′	
IκBβ	F:5′-TGATAGCAAGGTGAATGACGCTGTAG-3′	XM_015300227.1
	R:5′-CGGATGAGGTCGCAAGGCAAC-3′	
IKKα	F:5′-GAGGGGTGGAGGCTTAGATC-3′	NM_001012904.1
	R:5′-ACTTTCCTCGGGATGCAAGA-3′	
IKKβ	F:5′-TACAGGCAATCCAGACCTTCG-3′	NM_001031397.1
	R:5′-GACTGCCACTAACAGGACCAC-3′	
IKKε	F:5′-TGGATGGGATGGTGTCTGAAC-3′	XM_015299066.2
	R:5′-TGCGGAACTGCTTGTAGATG-3′	
TRAM	F:5′-GCCACGGTGTGGATACAAGT-3′	NM_001317736.1
	R:5′-ACATGCAACATCTTCGCCAC-3′	
TRAF6	F:5′-GAGTGTCCAAGGCGTCAAGTCTG-3′	XM_004941548.3
	R:5′-GTGTCGTGCCAGTTCATTCCTC-3′	
TBK1	F:5′-GGTTTGCCAGAATCGGAGT-3′	XM_015281552.2
	R:5′-TGTAAATACTCCTCTGTGCCGT-3′	
TAK1	F:5′-CCAGGAAACGGACAGCAGAG-3′	NM_001006240.2
	R:5′-GGTTGGTCCCGAGGTAGTGA-3′	
TRIF	F:5′-TCAGCCATTCTCCGTCCTCTTC-3′	NM_001081506.1
	R:5′-GGTCAGCAGAAGGATAAGGAAAGC-3′	
TRAF3	F:5′-CAGGATGCCACCTTCTCTCAC-3′	XM_004936341.3
	R:5′-AGGATGGTGTCGTTGAAGGAG-3′	
IRF3	F:5′-CGTATCTTCCGCATCCCTTGG-3′	NM_205372.1
	R:5′-TCGTCGTTGCACTTGGAGCG-3′	
IRF7	F:5′-GCCACGGTGTGGATACAAGT-3′	NM_001572.5
	R:5′-ACATGCAACATCTTCGCCAC-3′	

a 1 MyD88 = myeloid differentiation primary response protein 88; TIRAP/MAL = TIR, domain-containing adaptor protein/MyD88-adaptor-like; NF-κB = nuclear factor-kappa B; IκBα = NF-κB, inhibitor-alpha; IκBβ = NF-κB, inhibitor-beta; IKKα = IκB kinase complex-α; IKK-β = IκB kinase complex-β; IKK-ε = IκB kinase complex-ε; TRAM = TRIF-related adaptor molecule; TRAF6 = TNF-receptor-associated factor 6; TBK1 = tank-binding kinase1; TAK1 = transforming growth factor β-activated kinase 1; TRIF = TIR, domain-containing adaptor inducing IFN-β; TRAF3 = TNF-receptor-associated factor 3; IRF3 = IFN, regulatory factor 3; IRF7 = IFN, regulatory factor 7.

#### Whole Metagenomic Sequencing

1 broiler from each treatment group was randomly selected and slaughtered, DNA was extracted from the ileum content, and illumina TruSeq libraries were prepared from genomic DNA and sequenced on Illumina HiSeq systems 4,000 by Majorbio (China). Paired-end reads (2 × 150 bp) were generated, resulting in between 8 and 15 GB per sample (between 40 and 73 million paired reads). Briefly, to measure the abundance of known functional microbial genes in the ileum samples, reads from whole metagenomic sequencing were aligned to the Kyoto Encyclopedia of Genes and Genomes (KEGG) database using Majorbio (www.majorbio.com).

### Data Analysis

The experimental data were analyzed by the general linear model using SPSS 20.0 statistical package for Windows (2010, SPSS Inc., Chicago, IL 60606–6307). Difference and interaction among treatments were examined by one-way ANOVA with Duncan’s test. Calculated △Ct (corrected sample) = mean value of the target gene–mean value of the internal reference gene; △△ Ct = △Ct–mean value of the control group. Values of *p* < 0.05 mean a significantly statistical difference, and all the results were expressed as “mean ± standard deviation.”

## Results

### Effects of Quercetin on Growth Performance in AA Broilers

Compared with control, there were no significant differences in ADG, ADFI, and FCR among groups during the 42-day study (*p* > 0.05) (Table 6).

**TABLE 6:** Effect of quercetin on the growth performance of AA broilers.

Items	Control	Quercetin (%)		
		0.02	0.04	0.06
ADG (g/d)	59.75 ± 0.35	61.11 ± 1.04	62.03 ± 1.42	60.89 ± 1.06
ADFI (g/d)	100.08 ± 0.79	99.07 ± 0.33	100.40 ± 1.68	101.03 ± 1.04
FCR	1.67 ± 0.01	1.62 ± 0.02	1.62 ± 0.03	1.66 ± 0.04

a Data were statistically analyzed using one-way ANOVA, with Duncan’s test. Values are expressed as means ± S.D., and *n* = 6 for all groups.

b Values with different small superscript letters mean a significant difference (*p* < 0.05); Values with no letter or same superscript letters mean no significant difference (*p* > 0.05).

c ADG, average daily gain; ADFI, average daily feed intake; FCR, feed-to-gain ratio.

### Effects of Quercetin on Mortality Rate in AA Broilers

Maximum mortality rate (4.84%) was observed in the control group, and compared with control, dietary supplementation with 0.02%, 0.04, and 0.06% quercetin significantly decreased the mortality rate by 85.74%, 85.74, and 71.28%, respectively (*p* < 0.05, F = 9.06) (Table 7).

**TABLE 7:** Effect of quercetin on the mortality rate of AA broilers.

Items	Control	Quercetin (%)			*p* value	F value
		0.02	0.04	0.06		
Mortality rate	4.84 ± 0.05^a^	0.69 ± 0.69^b^	0.69 ± 0.69^b^	1.39 ± 0.88^b^	0.01	9.06

a Data were statistically analyzed using one-way ANOVA, with Duncan’s test. Values are expressed as means ± S.D., and *n* = 6 for all groups.

b Values with different small superscript letters mean a significant difference (*p* < 0.05).

### Effects of Quercetin on the mRNA Expression of Avian Beta-Defensins in the Ileum of AA Broilers

Dietary supplementation with 0.04 and 0.06% quercetin significantly upregulated the mRNA expression of total AvBD (*p* < 0.05, F = 4.27); however, quercetin did not significantly affect the mRNA expression of AvBD1 (*p* = 0.12, F = 2.22), AvBD2 (*p* = 0.49, F = 0.84), and AvBD14 (*p* = 0.43, F = 0.96) in the ileum of AA broilers, compared with control. In addition, 0.02% quercetin significantly upregulated the mRNA expression of AvBD3, AvBD4, AvBD6, and AvBD11 and downregulated the mRNA expression of AvBD12 and AvBD13 (*p* < 0.05). 0.04% quercetin significantly upregulated the mRNA expression of AvBD7, AvBD9, and AvBD10 (*p* < 0.05). 0.06% quercetin significantly upregulated the mRNA expression of AvBD5, AvBD8, AvBD10, AvBD11, and AvBD13 (*p* < 0.05). These results indicated that dietary quercetin supplementation regulated the mRNA expression of AvBD in the ileum of AA broilers (Table 8).

**TABLE 8:** Effect of quercetin on the mRNA expression of beta-defensins in the ileum of AA broilers.

Items	Control	Quercetin (%)			*p* value	F value
		0.02	0.04	0.06		
AvBD1	1.31 ± 0.43	0.92 ± 0.18	1.64 ± 0.20	2.01 ± 0.37	0.12	2.22
AvBD2	1.06 ± 0.17	1.43 ± 0.24	1.32 ± 0.33	0.92 ± 0.24	0.49	0.84
AvBD3	1.11 ± 0.20^a^	2.07 ± 0.26^b^	0.82 ± 0.29^a^	0.73 ± 0.11^ac^	0.00	7.22
AvBD4	1.11 ± 0.24^a^	2.91 ± 0.44^b^	2.15 ± 0.06^ab^	2.09 ± 0.59^ab^	0.11	2.26
AvBD5	1.11 ± 0.24^a^	0.84 ± 0.17^a^	1.26 ± 0.31^a^	2.33 ± 0.56^b^	0.04	3.42
AvBD6	1.10 ± 0.22^a^	2.15 ± 0.39^b^	1.82 ± 0.40^ab^	1.29 ± 0.21^ab^	0.11	2.31
AvBD7	1.10 ± 0.22^ab^	0.92 ± 0.13^b^	2.44 ± 0.37^c^	1.33 ± 0.21^ab^	0.00	7.43
AvBD8	1.13 ± 0.24^a^	1.79 ± 0.27^a^	1.52 ± 0.37^ab^	2.20 ± 0.40^b^	0.16	1.90
AvBD9	1.08 ± 0.18^a^	0.57 ± 0.07^a^	1.69 ± 0.33^b^	0.54 ± 0.08^a^	0.00	7.57
AvBD10	1.06 ± 0.16^ac^	0.57 ± 0.10^a^	2.77 ± 0.26^b^	2.27 ± 0.18^b^	0.00	30.61
AvBD11	0.99 ± 0.08^a^	1.90 ± 0.29^b^	0.63 ± 0.06^a^	2.73 ± 0.29^c^	0.00	20.10
AvBD12	1.03 ± 0.10^ac^	0.49 ± 0.06^b^	0.85 ± 0.13^ab^	1.47 ± 0.31^c^	0.00	5.16
AvBD13	1.06 ± 0.16^a^	0.55 ± 0.10^b^	0.78 ± 0.14^ab^	1.92 ± 0.19^c^	0.00	15.36
AvBD14	1.03 ± 0.10	0.73 ± 0.09	0.77 ± 0.13	0.87 ± 0.20	0.43	0.96
Total AvBD	15.28 ± 1.17^a^	17.83 ± 0.73^ab^	20.45 ± 1.95^bc^	22.71 ± 2.00^c^	0.02	4.27

a Data were statistically analyzed using one-way ANOVA, with Duncan’s test. Values are expressed as means ± S.D., and *n* = 6 for all groups.

b Values with different small superscript letters mean a significant difference (*p* < 0.05); values with no letter or same superscript letters mean no significant difference (*p* > 0.05).

### Effect of Quercetin on the Expression of Toll-Like Receptors in the Ileum of AA Broilers

Dietary supplementation with 0.02 and 0.06% quercetin significantly downregulated the mRNA expression of total TLR (*p* < 0.01, F = 33.5) in the ileum of AA broilers, compared with control. Dietary quercetin supplementation significantly downregulated the mRNA expression of TLR1A (*p* = 0.00, F = 28.85), TLR1B (*p* = 0.00, F = 42.40), and TLR2A (*p* = 0.00, F = 34.43); however, there were no significant differences in the mRNA expression of TLR2B (*p* = 0.06, F = 2.92) and TLR5 (*p* = 0.24, F = 1.54). Besides, 0.02% quercetin significantly downregulated the mRNA expression of TLR4 and TLR21 (*p* < 0.05); 0.04% quercetin significantly upregulated the mRNA expression of TLR4 and TLR7 (*p* < 0.05), and 0.06% quercetin significantly downregulated the mRNA expression of TLR3 (*p* < 0.05). These results indicated that dietary quercetin supplementation regulated the mRNA expression of TLR in the ileum of AA broilers (Table 9).

**TABLE 9:** Effect of quercetin on the mRNA expression of Toll-like receptors in the ileum of AA broilers.

Items	Control	Quercetin (%)			*p* value	F value
		0.02	0.04	0.06		
TLR1A	1.03 ± 0.10^a^	0.36 ± 0.03^b^	0.41 ± 0.04^b^	0.38 ± 0.03^b^	0.00	28.85
TLR1B	1.01 ± 0.07^a^	0.37 ± 0.04^b^	0.46 ± 0.04^b^	0.33 ± 0.04^b^	0.00	42.40
TLR2A	1.01 ± 0.06^a^	0.36 ± 0.06^b^	0.35 ± 0.03^b^	0.31 ± 0.06^b^	0.00	34.43
TLR2B	1.02 ± 0.10^ab^	0.75 ± 0.09^a^	1.24 ± 0.16^b^	0.97 ± 0.11^a^	0.06	2.92
TLR3	1.05 ± 0.15^a^	0.92 ± 0.08^a^	0.97 ± 0.13^a^	0.37 ± 0.03^c^	0.00	8.00
TLR4	1.03 ± 0.11^a^	0.39 ± 0.03^b^	1.92 ± 0.23^c^	1.16 ± 0.11^a^	0.00	20.27
TLR5	1.02 ± 0.09	1.15 ± 0.10	1.41 ± 0.21	1.50 ± 0.27	0.24	1.54
TLR7	1.04 ± 0.14^b^	1.10 ± 0.08^ab^	1.42 ± 0.18^a^	1.32 ± 0.09^ab^	0.15	2.00
TLR15	1.02 ± 0.09^ab^	0.83 ± 0.05^a^	1.23 ± 0.11^b^	1.13 ± 0.11^b^	0.03	3.52
TLR21	1.04 ± 0.12^a^	0.44 ± 0.03^b^	1.48 ± 0.31^a^	1.02 ± 0.17^a^	0.01	5.15
Total TLR	10.26 ± 0.35^a^	6.67 ± 0.32^b^	10.89 ± 0.31^a^	9.07 ± 0.38^c^	0.00	33.50

a . Data were statistically analyzed using one-way ANOVA, with Duncan’s test. Values are expressed as means ± S.D., and *n* = 6 for all groups.

b Values with different small superscript letters mean a significant difference (*p* < 0.05); values with no letter or same superscript letters mean no significant difference (*p* > 0.05).

### Effect of Quercetin on the Expression of Genes Related to the Toll-Like Receptor Signaling Pathway in the Ileum of AA Broilers

Dietary quercetin supplementation significantly downregulated the mRNA expression of MyD88 (*p* = 0.00, F = 15.98) and TIRAP/MAL (*p* = 0.00, F = 25.75); however, there were no significant differences in the mRNA expression of interferons regulatory factor 3 (IRF3) (*p* = 0.20, F = 1.68), TRIF, transforming growth factor β-activated kinase 1 (TAK1) (*p* = 0.20, F = 1.68), and TRAM (*p* = 0.38, F = 1.09) in the ileum of AA broilers, compared with control. 0.02% quercetin significantly downregulated the mRNA expression of tank-binding kinase1 (TBK1), nuclear factor-kappa B (NF-κB), NF-κB inhibitor-alpha (IκBα), IκBβ, IκB kinase complex-α (IKKα), IKKβ, IKKε, TNF-receptor-associated factor 3 (TRAF3), and IRF7 (*p* < 0.05), 0.04% quercetin significantly downregulated the mRNA expression of NF-κB, IκBα, IκBβ, IKKβ, IKKε, TRAF3, and TRAF6 (*p* < 0.05), and 0.06% quercetin significantly downregulated the mRNA expression of TBK1 and IKKα (*p* < 0.05). These results indicated that dietary quercetin supplementation regulated TLR signalling pathways and NF-κB signalling pathways in the ileum of AA broilers (Table 10).

**TABLE 10:** Effect of quercetin on the mRNA expression of genes related to the Toll-like receptor signaling pathway in the ileum of AA broilers.

Items	Control	Quercetin (%)			*p* value	F value
		0.02	0.04	0.06		
MyD88	1.01 ± 0.06^a^	0.35 ± 0.06^b^	0.64 ± 0.07^c^	0.41 ± 0.10^b^	0.00	15.98
TIRAP/MAL	1.03 ± 0.12^a^	0.44 ± 0.05^b^	0.20 ± 0.04^c^	0.28 ± 0.05^bc^	0.00	25.75
NF-κB	1.01 ± 0.06^a^	0.50 ± 0.05^b^	0.57 ± 0.11^b^	1.36 ± 0.25^a^	0.00	8.05
IκBα	1.04 ± 0.13^a^	0.58 ± 0.14^b^	0.52 ± 0.09^b^	0.77 ± 0.17^ab^	0.05	3.03
IκBβ	1.06 ± 0.16^a^	0.62 ± 0.06^b^	0.56 ± 0.06^b^	0.98 ± 0.08^a^	0.00	6.58
IKKα	1.05 ± 0.17^a^	0.53 ± 0.14^b^	0.91 ± 0.15^ab^	0.54 ± 0.10^b^	0.03	3.63
IKKβ	1.01 ± 0.06^a^	0.47 ± 0.04^b^	0.38 ± 0.04^b^	1.14 ± 0.19^a^	0.00	13.15
IKKε	1.01 ± 0.04^a^	0.48 ± 0.03^bc^	0.42 ± 0.06^b^	0.85 ± 0.27^ac^	0.02	4.17
TRAM	1.04 ± 0.13	1.26 ± 0.11	1.35 ± 0.08	1.30 ± 0.18	0.38	1.09
TRAF6	1.01 ± 0.07^a^	2.15 ± 0.37^ab^	3.50 ± 0.88^b^	0.78 ± 0.15^a^	0.00	6.67
TBK1	1.01 ± 0.06^a^	0.30 ± 0.04^b^	1.08 ± 0.10^a^	0.27 ± 0.03^b^	0.00	49.08
TAK1	1.01 ± 0.07^a^	1.74 ± 0.27^ab^	1.68 ± 0.26^ab^	1.79 ± 0.30^b^	0.61	0.62
TRIF	1.06 ± 0.17	1.39 ± 0.10	1.19 ± 0.09	1.15 ± 0.08	0.27	1.42
TRAF3	1.03 ± 0.11^a^	0.69 ± 0.14^b^	0.27 ± 0.05^c^	1.29 ± 0.09^a^	0.00	18.72
IRF3	1.04 ± 0.12	0.67 ± 0.06	0.75 ± 0.19	0.68 ± 0.14	0.20	1.68
IRF7	1.04 ± 0.13^a^	0.68 ± 0.25^b^	0.69 ± 0.08^a^	0.84 ± 0.06^a^	0.00	8.26

a Data were statistically analyzed using a one-way ANOVA, with Duncan’s test. Values are expressed as means ± S.D., and *n* = 6 for all groups.

b Values with different small superscript letters mean a significant difference (*p* < 0.05); values with no letter or same superscript letters mean no significant difference (*p* > 0.05).

### Effect of Quercetin on Microbial Diversity in the Ileum of AA Broilers

**FIGURE 1:**
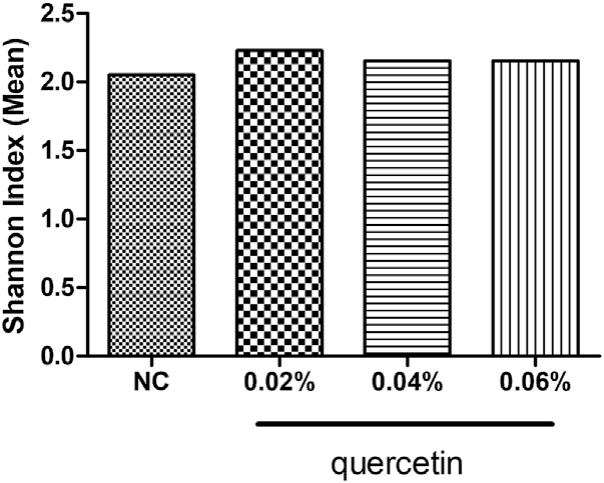
Effect of quercetin on microbial diversity in the ileum of AA broilers, NC (normal control broilers) and quercetin (0.02%, 0.04, and 0.06%) groups. Data were statistically analyzed using one-way ANOVA with Duncan’s test. Values are expressed as means ± S.D., and *n* = 1 for all groups. Values not marked mean no significant difference was seen (*p* > 0.05).

Quercetin had no significant effect on the Shannon index in the ileum of AA broilers (*p* > 0.05) (Figure 1).

### Effect of Quercetin on the Relative Abundance of Microbes in the Ileum of AA Broilers at the Genus and Family Level

**FIGURE 2:**
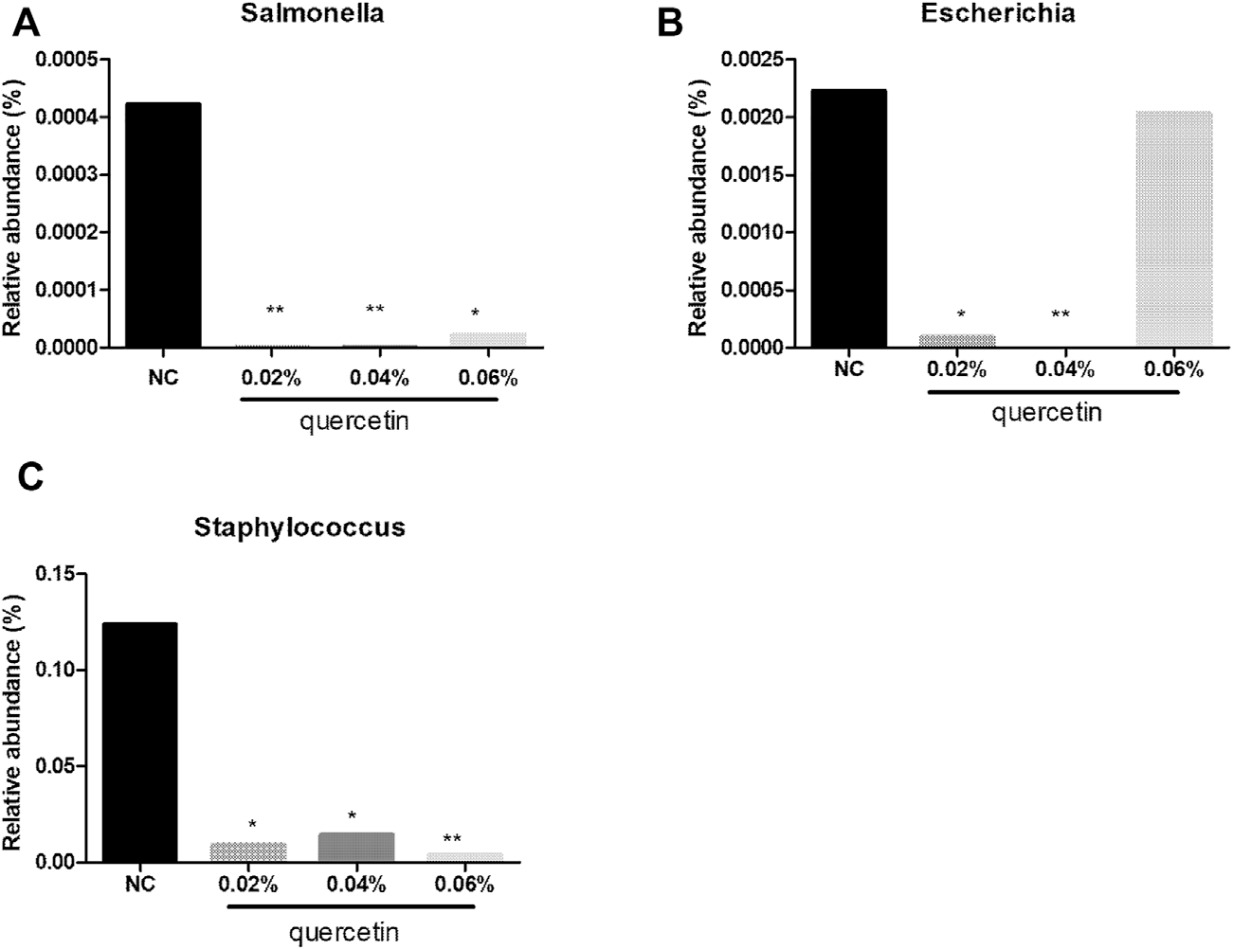
Effect of quercetin on microbial relative abundance in the ileum of AA broilers at the genus level, NC (normal control broilers) and quercetin (0.02%, 0.04, and 0.06%) groups. **(A)** Effect of quercetin on Salmonella relative abundance in the ileum of AA broilers at the genus level; **(B)** Effect of quercetin on Escherichia relative abundance in the ileum of AA broilers at the genus level; **(C)** Effect of quercetin on Staphylococcus relative abundance in the ileum of AA broilers at the genus level. Data were statistically analyzed using one-way ANOVA with Duncan’s test. Values are expressed as means ± S.D., and *n* = 1 for all groups. *p < 0.05 and **p < 0.01.

At the genus level, compared with control, 0.02% quercetin significantly decreased the relative abundance of *Escherichia*, *Staphylococcus* (*p* < 0.05), and *Salmonella* (*p* < 0.01), 0.04% quercetin significantly decreased the relative abundance of *Staphylococcus* (*p* < 0.05), *Escherichia*, and *Salmonella* (*p* < 0.01), and 0.06% quercetin significantly decreased the relative abundance of *Salmonella* (*p* < 0.05) and *Staphylococcus* (*p* < 0.01) in the ileum of AA broilers (Figure 2).

**FIGURE 3:**
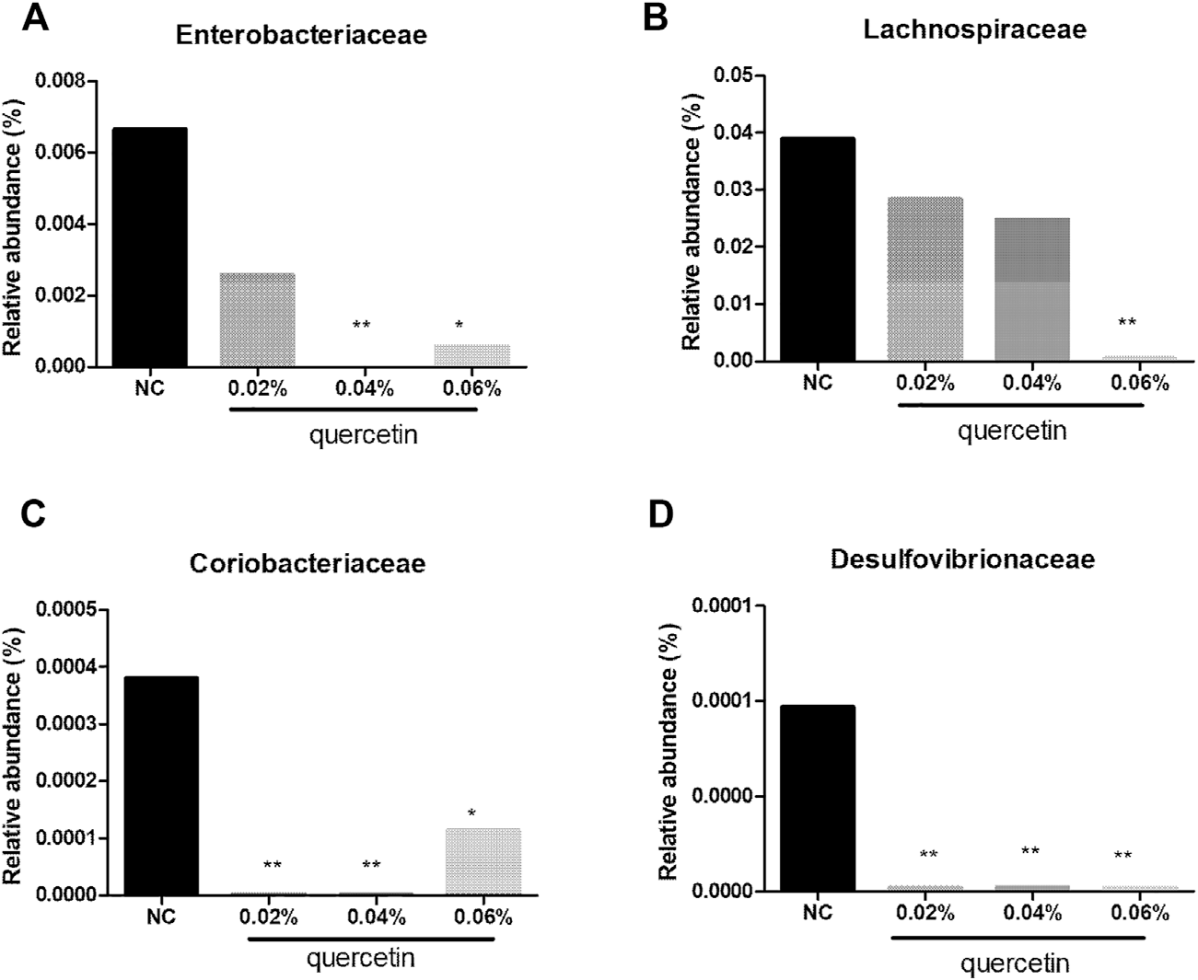
Effect of quercetin on microbial relative abundance in the ileum of AA broilers at the family level, NC (normal control broilers) and quercetin (0.02%, 0.04, and 0.06%) groups. **(A)** Effect of quercetin on Enterobacteriaceae relative abundance in the ileum of AA broilers at the family level; **(B)** Effect of quercetin on Lachnospiraceae relative abundance in the ileum of AA broilers at the family level; **(C)** Effect of quercetin on Coriobacteriaceae relative abundance in the ileum of AA broilers at the family level; **(D)** Effect of quercetin on Desulfovibrionaceae relative abundance in the ileum of AA broilers at the family level. Data were statistically analyzed using one-way ANOVA with Duncan’s test. Values are expressed as means ± S.D., and *n* = 1 for all groups. *p < 0.05 and **p < 0.01.

At the family level, compared with control, 0.02% quercetin significantly decreased the relative abundance of Coriobacteriaceae and Desulfovibrionaceae (*p* < 0.01), 0.04% quercetin significantly decreased the relative abundance of Enterobacteriaceae, Coriobacteriaceae, and Desulfovibrionaceae (*p* < 0.01), and 0.06% quercetin significantly decreased the relative abundance of Enterobacteriaceae, Coriobacteriaceae (*p* < 0.05), Lachnospiraceae, and Desulfovibrionaceae (*p* < 0.01) in the ileum of AA broilers (Figure 3).

### Estimate of the Optimal Supplementation of Dietary Quercetin in AA Broilers

Through the quadratic curve regression model, the quadratic regression equation of quercetin supplementation level and mortality, total AvBD, and total TLR expression in AA broiler diets was established, and the optimal range of quercetin supplementation was calculated by considering the following data comprehensively. The optimum addition amount of quercetin is 0.04% under the test conditions (Table 11).

**TABLE 11:** Optimal quercetin level in AA broilers on the fitting curve.

Dependent variable	Regression equation	Coefficient	*p* value	Optimal level of quercetin%
Mortality rate	y = 1836.50x^2^−162.17x+3.91	0.37	0.01	0.044
Total AvBD	y = −116.77x^2^+108.32x+15.26	0.39	0.02	0.119
Total TLR	y = −46.80x^2^+11.22x+0.81	0.12	0.00	0.463
MyD88	y = 172.38x^2^−18.974x+0.9358	0.42	0.00	0.055
NF-κB	y = 520.22x^2^−34.501x+1.0146	0.55	0.00	0.042
*Salmonella enterica*	y = 0.21x^2^−0.01x+7e^−5^	0.94	0.00	0.025
*Escherichia coli*	y = 2.24x^2^−0.19x+3.6e^−3^	0.99	0.00	0.040
*Staphylococcus aureus*	y = 24.875x^2^−2.5555x+0.0585	0.94	0.00	0.005

## Discussion

The present study showed that dietary quercetin supplementation significantly upregulated the mRNA expression of total AvBD and significantly downregulated the mRNA expression of total TLR and regulated the TLR signaling pathway in the ileum of AA broilers (*p* < 0.05). The current study was the first report regarding the effects and mechanism of quercetin on the mRNA expression of AvBD in the ileum of AA broilers.

Flavonoids exhibited different effects on growth in chicken. Dietary supplementation with 0.02%, 0.04, and 0.06% quercetin had no significant effect on ADG, ADFI, and FCR in AA broilers during the 42d study (*p* > 0.05). Similar results were reported by Sayed et al. (2015), who studied 10%, 20, and 30% buckwheat (rich bioactive compounds including flavones and flavonoids) supplementation for 4 weeks had no effect on body weight gain, feed intake, and FCR (*p* > 0.05) in broilers. However, these results were different from those of Hassan et al. (2018), who found that dietary rutin supplementation with 1 g/kg diet for 42d significantly increased body weight gain and decreased FCR (*p* < 0.001), and there were no linear or quadratic effects on either feed intake or the relative growth rate (*p* > 0.05). The variation resulted from the difference in types of flavonoids and experimental animals. Gut microbiota is composed of trillions of bacteria, which play an important role in animal metabolism. Desulfovibrioceae, *Rhodospirillum*, and *Lacetospirillum* are positively correlated with obesity in mice, and quercetin and resveratrol reduced the relative abundance of these bacteria (Zhao et al., 2017). The previous research of our lab found that quercetin reduced the relative abundance of *Desulfovibrio desulphuricans*, Miridae, and Spirilloidea in the ileum of broilers (data not shown) and the blood lipid content of AA broilers, increased the secretion of leptin and adiponectin, activated the phosphatidylinositol 3-kinase/protein kinase B (PI3K/PKB) signaling pathway, thereby improving lipid metabolism, and ultimately reduced the abdominal fat deposition in broilers (Ying et al., 2020). Combined with the results of this study, it was indicated that quercetin improved lipid metabolism and reduced fat deposition of broilers, which may result from regulating some of the obesity-related flora in the intestine. That is, quercetin interfered with lipid metabolism by reducing the abundance of intestinal flora such as Laospirillaceae, which is positively related to obesity, thereby reduced fat deposition, and then, played an antiobesity role. This also explained why quercetin has no significant effect on the growth performance of AA broilers.

The decrease of mortality was observed in broilers supplemented with quercetin (*p <* 0.05), and similar results were reported by Kalia et al. (2018), who examined the effects of *H. rhamnoides* aqueous extract (100, 150, 200, 300, 400, and 800 mg/kg body weight for 42d) on the mortality of broilers, which is abundant in bioactive plant molecules including flavonoids, vitamins, carotenoids, and organic acid. Fan et al. (2019) reported that rabbit neutrophil peptide-1 (NP-1) is a multifunctional cationic peptide with broad-spectrum antimicrobial activities, which increased duodenal *Lactobacillus* and adjusted the balance and quality of the intestinal microflora, thus improving chicken health. In birds, AvBDs are the biggest cluster of host defense peptide (HDPs), and HDPs potentially control a wide range of pathogens without resistance; moreover, they play important roles in adaptive immunity, wound healing, and sperm fertilization and may also be used as alternatives to antibiotics (Lynn et al., 2007; Hazlett and Wu, 2010; Zhang et al., 2016). Fourteen kinds of AvBDs are present in chickens which play an important role in the innate host defence against viruses, Gram-positive and Gram-negative bacteria, and parasites in the digestive system, reproductive system, and immune function in poultry (Lynn et al., 2007; Zhang and Sunkara, 2014; Cheng et al., 2015). AvBD is an effective tool for maintaining health and reducing the burden of infectious disease in livestock species (Meade and O’Farrelly, 2019). Infectious bronchitis virus (IBV) infection induces the mRNA expression of AvBD2, 4, 5, 6, 9, and 12, which suggests that AvBDs may play significant roles in resisting IBV replication in chickens (Xu et al., 2015). There are few studies that focus on the flavonoids in promoting AvBD expression. The black tea extract and the aflavin derivatives induced the secretion of the antimicrobial peptides human β-defensin 1(hBD-1), hBD-2, and hBD-4 in oral epithelial cells to protect the oral cavity against periodontal disease (Bedran et al., 2013). *Zizania latifolia* has been used as a health food in Asian countries, which may upregulate the mRNA expression of hBD in HaCaT cells and, thus, inhibit *S. aureus* infection through the TLR2 signaling pathway (Kang et al., 2018). In the present study, compared with control, dietary supplementation with 0.04 and 0.06% quercetin significantly upregulated the mRNA expression of total AvBD (*p* < 0.05) in the ileum of AA broilers. The high expression of AvBD6 in the proximal digestive tract and broad antimicrobial activity indicate that AvBD6 plays an important role in chicken innate host (Van Dijk et al., 2006). The present results showed that dietary quercetin supplementation significantly upregulated the mRNA expression of AvBD10 (0.04 and 0.06%, *p* < 0.05) and AvBD11 (0.02 and 0.06%, *p* < 0.05), and these findings are consistent with the results of Laptev et al. (2019); it was shown that phytobiotic intebio enriched with essential oils (90 g/t for 42d) significantly upregulated the mRNA expression of AvBD10 (*p* < 0.05) in the early stage of *Salmonella* infection (at 1 dpi); however, in the late stage of *Salmonella* infection (23dpi), the mRNA expression of AvBD11 was significantly lowered, and it suggested that the essential oils inhibited the earlier inflammatory reaction. Hong et al. (2012) demonstrated that AvBD10 induction was detected in response to *Salmonella* of poultry. In the current study, compared with control, dietary quercetin supplementation did not influence the mRNA expression of AvBD1, AvBD2, AvBD8, and AvBD14 (*p* > 0.05); however, dietary quercetin supplementation significantly upregulated the mRNA expression of AvBD3, AvBD4, AvBD5, AvBD6, AvBD7, AvBD8, AvBD9, AvBD10, AvBD11, and AvBD13 (*p* < 0.05) and significantly downregulated the mRNA expression of AvBD12 (*p* < 0.05). Lee et al. (2016) reported synthetic linear AvBD8 and AvBD10 peptides have weak lytic activity against *Escherichia coli* (Semple and Dorin, 2012). The abovementioned results revealed that quercetin reduced the mortality rate of broilers by promoting the expression of intestinal AvBDs, thereby promoting the health of broilers.

TLRs are type I trans-membrane proteins present either on the cell surface or in the intracellular compartments and the important components of the innate immune system, which are responsible for recognizing and responding to microbial components and endogenous molecules that are released by metabolism (Jin and Lee, 2008; Kang and Lee, 2011). Ten kinds of TLRs in chicken have been identified since the first report of the Toll protein in fruit fly (Anderson et al., 1985). There are two types of TLR signaling pathways, including the MyD88-dependent pathway and the TRIF-dependent pathway, respectively. Almost all the TLRs are activated in the MyD88-dependent response except for TLR3 (Kawai et al., 1999; Takeuchi et al., 2000; Kawai and Akira, 2007; Keestra et al., 2013; Warden et al., 2018). Flavonoids may regulate the expression of the TLR and TLR signaling pathway. Nawab et al. (2019) reported that curcumin downregulates the gene expression of TLR4 and associated downstream molecules such as NF-κB, MyD88, and TNF-α in laying hens. EGCG is the most abundant polyphenol in green tea, accounting for more than 50% of total polyphenols, and it effectively attenuated the acute lung injury induced by paraquat pesticides (PQ) via inhibiting the activation of NF-κB and upregulating TLR 2, 4, and 9 as well as their adaptors MyD88 and TRAF6 in mice (Oliveira et al., 2016; Shen et al., 2017). Quercetin regulated the TLR signaling pathway and inhibited NF-κB activity only via TAK1 in a dose-dependent manner, thus alleviating neuropathic pain (Ji et al., 2017). Cheng et al. (2017) showed that baicalin downregulated the expression of TLR4 and inhibited NF-κB activation in lipopolysaccharide (LPS)-induced liver inflammation of chicken via negatively regulating inflammatory mediators. The current study showed that dietary supplementation with 0.02%, 0.04, and 0.06% quercetin significantly downregulated the mRNA expression of TLR1A, TLR1B, TLR2A, and total TLR in the ileum of AA broilers (*p* < 0.05). In addition, the mRNA expression of TLR4 and TLR21 (0.02% quercetin supplementation, *p* < 0.05) and TLR3 (0.06% quercetin supplementation, *p* < 0.05) was significantly downregulated, and 0.04% quercetin significantly upregulated the mRNA expression of TLR4 and TLR7 (*p* < 0.05). Dietary quercetin supplementation effectively prevented inflammation via inhibiting the activation of NF-κB and downregulating total TLR, TLR1A, TLR1B, TLR2A, and TLR2B2, as well as their adaptors MyD88 and TIRAP/MAL (*p* < 0.05), in the ileum of AA broilers. 0.02% quercetin significantly downregulated the mRNA expression of TBK1, NF-κB, IκBα, IκBβ, IKKα, IKKβ, IKKε, TRAF3, and IRF7 (*p* < 0.05); 0.04% quercetin significantly downregulated the mRNA expression of NF-κB, IκBα, IκBβ, IKKβ, IKKε, TRAF3, and TRAF6 (*p* < 0.05), and 0.06% quercetin significantly downregulated the mRNA expression of TBK1 and IKKα (*p* < 0.05). The previous research of our lab found that dietary quercetin supplementation significantly increased the mRNA expression of TNF-α, TRAF2, nuclear factor-kappa-Bp65 subunit (NF-κBp65), and IFNγ (*p* < 0.05) and decreased the mRNA expression of IκBα (*p* < 0.05); that is, quercetin improved immune function via the NF-κB signaling pathway triggered by TNF-α (Yang et al., 2020). These results indicated that flavonoids played an anti-inflammatory role by inhibiting the expression of TLR and signal molecules related to TLR signaling pathways and NF-κB signalling pathways, thereby improving immunity and promoting health. The inflammatory process is a potential life-saving response to microbial challenge, which is ultimately to prevent tissue and the host from damage (Swaggerty et al., 2016). Under commercial farming models, broilers are exposed to a series of potential pathogens, with higher mRNA expression of proinflammatory factors in peripheral blood neutrophils, resisting necrotic enteritis induced by *Salmonella*, *Eimeria tenella*, and *Clostridium perfringens* (Swaggerty et al., 2014; Swaggerty et al., 2015). All the results showed that the inflammatory response plays an important role in enhancing the resistance of chicken to common pathogen infections, and a moderate inflammatory response is beneficial to the health of poultry.

**FIGURE 4:**
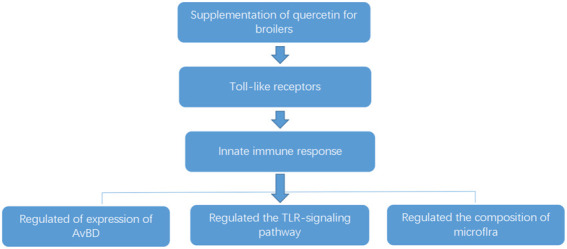
The potential mechanism of quercetin on the morality rate of broilers.

The previous studies in our lab found that quercetin significantly regulated cecal flora of chickens. Wang found that quercetin reduced the number of *Pseudomonas aeruginosa*, *Helicobacter pylori*, *Salmonella*, *Staphylococcus aureus*, *Escherichia coli*, *Clostridium perfringens*, and *Campylobacter jejuni* in cecum of AA broilers (Wang, 2018). Quercetin also increased the number of bifidobacteria and reduced the abundance of *Escherichia coli*, *Staphylococcus aureus*, *Salmonella*, *Pseudomonas aeruginosa*, *Streptococcus faecalis*, and *Enterococcus* in cecum of laying hens (Teng, 2015). In addition, quercetin reduced the number of the total *Escherichia coli* and increased the number of Bifidobacteria and *Lactobacillus* in cecum of laying hens (Jin, 2013). In the present study, 0.02% quercetin significantly decreased the relative abundance of *Escherichia*, *Staphylococcus* (*p* < 0.05), and *Salmonella* (*p* < 0.01), 0.04% quercetin significantly decreased the relative abundance of *Staphylococcus* (*p* < 0.05), *Escherichia*, and *Salmonella* (*p* < 0.01), and 0.06% quercetin significantly decreased the relative abundance of *Salmonella* (*p* < 0.05) and *Staphylococcus* (*p* < 0.01) in the ileum of AA broilers. Together with the abovementioned results, quercetin significantly regulated the intestinal flora of chickens, which reduced the colonization of harmful bacteria and promoted the colonization of beneficial bacteria in the intestinal tract, thereby improving the microecological balance of the intestinal tract, thus promoting the healthy growth of broilers. The potential effects of quercetin on reducing the morality rate of broilers mediates the innate immune responses by modulating the expression of TLRs, and there are three primary levels: 1) by regulating the expression of AvBD, 2) by regulating the TLR signaling pathways, and 3) by adjusting the composition of microbiota (Figure 4).

## Conclusion

In conclusion, quercetin enhances immunity by regulating the expression of AvBD, TLRs, and the TLR signaling pathways and NF-κB signalling pathways, to achieve the purpose of anti-inflammatory effect, and maintains the balance of intestinal flora by modulating the composition of the microbiota, so as to promote the body health and reduced mortality rate of broilers, and the optimum addition amount of quercetin is 0.04% under the test conditions.

## Data Availability

Publicly available datasets were analyzed in this study. This data can be found here: https://www.ncbi.nlm.nih.gov/bioproject/808675/BioProject ID PRJNA808675. The original contributions presented in the study are publicly available. This data can be found here: https://www.ncbi.nlm.nih.gov/biosample/26101061/SAMN26101061, https://www.ncbi.nlm.nih.gov/biosample/26101062/SAMN26101062, https://www.ncbi.nlm.nih.gov/biosample/26101063/SAMN26101063, https://www.ncbi.nlm.nih.gov/biosample/26101064/SAMN26101064, https://www.ncbi.nlm.nih.gov/biosample/26101065/SAMN26101065, https://www.ncbi.nlm.nih.gov/biosample/26101066/SAMN26101066, https://www.ncbi.nlm.nih.gov/biosample/26101067/SAMN26101067, https://www.ncbi.nlm.nih.gov/biosample/26101068/SAMN26101068, https://www.ncbi.nlm.nih.gov/biosample/26101069/SAMN26101069, https://www.ncbi.nlm.nih.gov/biosample/26101070/SAMN26101070, https://www.ncbi.nlm.nih.gov/biosample/26101071/SAMN26101071, https://www.ncbi.nlm.nih.gov/biosample/26101072/SAMN26101072.
